# Case of Diseased Liver, Illustrative of the Necessity of Attending to the More Important Organs in Disease, and Interesting on Account of the Symptoms

**Published:** 1814-05

**Authors:** H. H. Tullidge

**Affiliations:** Member of the Royal College of Surgeons, London


THE
### Medical and Physical Journal.

5 OF VOL. XXXI.] MAY, 1814. [NO. 183.

" For many fortunate discoveries in medicine, and for the detection of nume-
" rous errors, the world is indebted to the rapid circulation of Monthly
" Journals; and there never existed any work to which the faculty in
" EUROPE and AMERICA were under deeper obligations than to the
" Medical and Physical Journal of London, now forming a long, but an
" invaluable series."—RUSH.

### *For the Medical and Physical Journal.*

*Case of Diseased Liver, illustrative of the necessity of attending to the more important Organs in Disease, and interesting on account of the Symptoms; by* Mr. H. H. TULLIDGE, Member of the Royal College of Surgeons, London.

MR. B., aged thirty-four, a merchant of this town, was attacked very frequently during the summer with a troublesome hiccup, which at first was not much attended to, but as it became more frequent and of longer duration, from two to six hours and upwards, he applied to a medical gentleman, who prescribed for him various remedies, but with little or no effect. His health in a few weeks was manifestly on the decline, with great loss of muscular energy. The beginning of October his hiccup was so incessant as only to have a respite of half an hour or an hour in twenty-four. At this time I was sent for. On making my inquiries, I found his bowels for some time past had been unusually costive, so as to render opening medicine often necessary: he had observed also that he made less water; he complained of weakness across the loins, and, what is remarkable, a total insensibility of the scalp, so much so that no sensation whatever was excited by pulling the hair ever so strong; his pulse at this time were by no means frequent, but easily compressible; he complained of no local pain of any kind. I first gave him powders composed of the Pulv. Rhei. cm. Sulphate Potassæ, with small doses of the Pulv. Ipecac. every two hours, with a view to excite some little action on the stomach, and to clear the primæ viæ. Slight vomiting was brought on by the second dose of the powders, but no action was produced on the bowels, the hiccup however continuing. Having maturely considered the case, though the cause of the incessant hiccup was obscure, it appeared evident from the the other symptoms there was a want of action in the secreting system, and of the liver particularly. With this view of the case, he was ordered the Pil. Hydrarg. cum. Opio et Ipecac. every four hours, expecting that the combined effects of the opium and ipecacuanha would have some effect in relieving the hiccup, and produce a general moisture of the skin, and that the hydrargyrus would exert its influence on the secreting system. On account of indisposition, I could not see him for a few days after this, but a medical friend called at my request, who reported to me that the hiccup had remitted between two and three hours, that he had some sleep, his bowels were relieved, and his pulse was favorable. The pills were recommended to be continued. Another practitioner, however, at this time was called in, who considering the symptoms to arise from renal calculi passing the ureter, strongly recommended the alkalies, which were resorted to, and continued until the case was hopeless, and he died.

The friends of Mr. B. requested that the body might be opened, to ascertain if possible the nature of his complaint. I accordingly attended, and in the presence of several surgeons opened the body. The first mark of disease which presented to us on exposing the abdominal viscera, was a dark ridge on the margin of the whole of the right lobe of the liver; and on raising the sternum to bring into view the whole of the convex surface of this viscus, the posterior and middle surface was of a black grumous appearance, which, on cutting into, was found to enter some depth into its substance. The gall-bladder was empty, and no trace of bile could be distinguished in any of the pori biliarii. The renales were examined, but no mark of disease was apparent. The ureters were free from any obstruction. The bladder was much contracted, and contained little or no urine. There was no diseased appearance in any of the other viscera.

Reflecting on the symptoms and the cause of the hiccup, contrasted with a view of the principal seat of disease on dissection, it appears to me that the hiccup was induced by the diaphragm being opposed to the inflamed surface of the liver. The absence of pain in the hepatic region, and the insensibility of the scalp complained of by the patient, is still somewhat obscure.

*St. John's, Newfoundland,* H. H. TULLIDGE.
*Nov.* 10, 1813.

